# Psychological profiles of anti-vaccination argument endorsement

**DOI:** 10.1038/s41598-023-30883-7

**Published:** 2023-07-17

**Authors:** Dawn L. Holford, Angelo Fasce, Thomas H. Costello, Stephan Lewandowsky

**Affiliations:** 1 School of Psychological Science, University of Bristol, Bristol, BS8 1TU UK; 2 University of Coimbra, 3004-531 Coimbra, Portugal; 3 Massachusetts Institute of Technology, Cambridge, MA 02139 USA

**Keywords:** Human behaviour, Health care

## Abstract

The proliferation of anti-vaccination arguments online can threaten immunisation programmes, including those targeting COVID-19. To effectively refute misinformed views about vaccination, communicators need to go beyond providing correct information and debunking of misconceptions, and must consider the underlying motivations of people who hold contrarian views. Drawing on a taxonomy of anti-vaccination arguments that identified 11 “attitude roots”—i.e., psychological attributes—that motivate an individual’s vaccine-hesitant attitude, we assessed whether these attitude roots were identifiable in argument endorsements and responses to psychological construct measures corresponding to the presumed attitude roots. In two UK samples (total *n* = 1250), we found that participants exhibited monological belief patterns in their highly correlated endorsements of anti-vaccination arguments drawn from different attitude roots, and that psychological constructs representing the attitude roots significantly predicted argument endorsement strength and vaccine hesitancy. We identified four different latent anti-vaccination profiles amongst our participants’ responses. We conclude that endorsement of anti-vaccination arguments meaningfully dovetails with attitude roots clustering around anti-scientific beliefs and partisan ideologies, but that the balance between those attitudes differs considerably between people. Communicators must be aware of those individual differences.

## Introduction

Vaccinations are one of the most successful medical inventions for controlling and preventing deaths from infectious diseases^1^. Curiously, however, opposition to vaccines remains prevalent and therefore poses a substantial threat to global health^2^. In particular, the proliferation of anti-vaccination arguments on the Internet has curtailed the benefits of many immunisation programmes^3,4^—with COVID-19 vaccinations offering an especially salient recent example^5^. These arguments influence individuals’ decisions to have vaccinations. Indeed, mere exposure to online vaccine misinformation may lower vaccination intentions^6^ and belief in misinformation is cross-culturally associated with lower readiness to be vaccinated against COVID-19^7^. The perpetuation of misconceptions and logical fallacies by vaccine opponents have also influenced the intentions of parents to vaccinate their children^8,9^.

Vaccine-hesitant individuals express arguments that can range expansively from exaggerated safety concerns, to the use of fallacious logic, to reliance on misinformation, to conspiratorial beliefs, to give some examples^10^. This can make it difficult for vaccine communicators—such as fact-checkers, healthcare professionals, and scientists—to counter the many different arguments that spread rapidly on the Internet^11^. Compounding the problem, facts and evidence to debunk flawed contrarian argumentation may not be sufficient. Opposition to vaccines that stems from social and cultural factors, rather than a failure to understand the science of vaccination, will not necessarily be satisfactorily countered with scientific evidence^12^. Certain anti-vaccination arguments also target cognitive systems that are used in intuitive judgements and motivated reasoning, which can make it harder to combat those arguments with statistics, facts, and logic^13^. Further, people may be motivated to reject scientific evidence if it is in conflict with their personal interests, worldviews, or beliefs^14,15^. In those cases, people may engage in motivated reasoning such that they interpret scientific findings in a manner that is compliant with their existing beliefs^16^. For example^17^, found that people evaluated information compatible with their existing attitudes about flu vaccination to be more convincing than attitude-inconsistent information.

Effective rebuttal of anti-vaccination arguments therefore requires an approach that goes beyond addressing flaws in the arguments, by also considering the underlying psychological attributes, known as “attitude roots”^18,19^, that drive opposition to vaccines. This means looking beyond the content of arguments to assess what motivates someone to endorse an anti-vaccination argument. Such motivations could be based on very different psychological constructs, including “fears, ideologies, worldviews, and identity needs”^18^. For instance, individuals high in conspiratorial ideation (a psychological tendency) may tend to argue that one should reject vaccinations because they are part of a secret plot to control the population by implantation of microchips embedded in the vaccines, whereas individuals who are politically libertarian (a worldview) may argue that one should reject vaccinations because they are a political tool that removes people’s freedoms (e.g., through mandates). Ultimately, the attitude roots identifiable in the expression of an argument should serve as a veneer for stable individual differences (e.g., personality, values, worldviews, or emotions) that are also related to vaccine hesitancy.

Understanding the attitude root of an individual’s resistance to vaccines may thus allow vaccine communicators to align their message with the individual’s motivation for holding their position, and avoid triggering their motivation to reject the pro-vaccination message^18,20^. However, identifying an attitude root is no easy task. As the terminology suggests, attitude roots lie beneath a surface expression and are not always obvious to the interlocutor. Individuals may themselves lack insight into their own motivations for endorsing a particular anti-vaccination argument^18^. Further complicating matters, even the manifestations of attitude roots can overlap. As seen in the example above, a secret plot to control the population will also remove people’s freedoms. Therefore, to better understand how to address the attitude roots of vaccine opposition, there is a need for research to investigate the manifestations of anti-vaccination sentiment (i.e., arguments) and link those manifestations to underlying psychological factors. Earlier work showed that across 24 different countries, three psychological factors (conspiratorial beliefs, disgust about blood and injections, and reactance) were associated with negative attitudes about the safety and effectiveness of children’s vaccinations, suggesting that these could be potential attitude roots to investigate as motivations to reject vaccination science^20^. More recent work^21^ sought to classify a wider range of anti-vaccination arguments and map them to potential attitude roots. In this work, the authors identified 2414 anti-vaccination arguments through a PRISMA-compliant systematic review of 152 scientific publications, and classified them into a hierarchical taxonomy with 11 overarching attitude roots. This classification, initially done by qualitative thematic analysis, was validated using machine learning to classify arguments based on their linguistic expression. Trained researchers classified the attitude roots in two different datasets—the arguments obtained from the systematic literature review, and an additional dataset of 582 anti-vaccination arguments obtained from a database of fact-checked COVID-19 vaccine claims circulating on the Internet. A Natural Language Processing model trained on a subset of the data was able to predict the attitude root classifications with a high level of accuracy.

This taxonomy^21^ integrated decades of prior research on the typologies of anti-vaccination arguments (e.g.,^10,22^). It conceptualised anti-vaccination arguments, which form the base level of the taxonomy, as an expressed proposition that opposes vaccination—i.e., the given reason for not having a vaccine. The 11 attitude roots that form its top level (see Table 1) reflect psychological characteristics that have been found in past research to be related to vaccine hesitancy (e.g., conspiracist beliefs^23,24^).

**Table 1:** Attitude roots identified in a taxonomy of anti-vaccination arguments.

Attitude root	Description	Associated psychological construct
Conspiracist ideation	A tendency to believe in, or assume that, a complex causal chain of secret events exists when there are other, more probable, explanations for phenomena	Conspiracy mentality
Distrust	A general mistrust in the sources of information about vaccines, as well as a perception that these sources have vested interests or lack knowledge. Distrust is related to conspiracist ideation, but arguments expressing distrust tend to manifest as vague statements of suspicion or uncertainty without proposing the existence of a conspiracy	General distrust
Unwarranted beliefs	A variety of beliefs that lack or misrepresent scientific evidence or facts, or are based on pseudoscientific doctrines—for example, that naturopathic treatments are more effective than scientific medicine	Pseudoscientific beliefs
Worldview and politics	Individuals’ perspectives on the way society should be organised, encompassing worldviews and political ideologies such as populism, nationalism, and conservatism	
Religious concerns	A range of religious or spiritual beliefs and norms, including concerns about diet, purity, and a perceived natural order, and beliefs in religious alternatives to healing	Centrality of religion
Moral concerns	Perceptions that vaccinations are promoting behaviour or acts that the individual considers immoral, which may, but need not be related to one’s religious beliefs—for example, individuals may oppose abortion for moral reasons without appealing to religious beliefs, even if the two are often related	Moral absolutism
Fears and phobias	Different fears about vaccines that are typically disproportionate to the actual dangers. Fears can be of the perceived consequences from vaccination, or of the vaccination procedure itself	Trait fear
Distorted risk perception	A lack of fear or awareness about the threat posed by the disease, leading to a distorted risk-benefit calculation about vaccination based on the misperceived risks	Perceived vaccination risk
Perceived self-interest	A prioritisation of one’s individual needs over those of others, reflecting a lower level of collective responsibility	Prosocial behavioural intentions
Epistemic relativism	A view that “truth” is only a social convention and therefore scientific expertise, evidence, and facts should not be placed on a higher footing (or should be downplayed) relative to subjective experiences and intuitions	Alternative epistemology
Reactance	A tendency for individuals to defend their autonomy when they perceive that their freedoms are being restricted or that others are trying to impose their will on them	General reactance

All attitude roots are directly adopted from^21^. See text in “Methods” for description of the scales used to measure the associated psychological construct.

Fasce et al.’s^21^ taxonomy provided the most comprehensive framework to date of a wide range of arguments and their links to the underlying attitude roots of anti-vaccination belief—i.e., their psychological motivators. We used the taxonomy as a springboard for the present investigation of the psychological factors that motivate people’s anti-vaccination attitudes. We investigated these attitude roots in two ways. First, we sought to assess whether it would be possible to observe clusters of argument endorsement that reflect correlated levels of endorsements for anti-vaccination arguments within the same attitude root. Of course, the boundary between attitude roots may be blurred, with overlaps between those that share similarities (such as religious and moral concerns^21^). An individual could also hold more than one attitude root, thus strengthening their motivation to endorse anti-vaccination arguments^19^. Indeed, there is evidence from research into conspiracist beliefs that individuals may form monological belief systems, where belief in one conspiracy theory supports belief in others^25,26^.

Our second goal was to determine if argument endorsements were associated with specific psychological characteristics that were identified as individual difference measures for the attitude roots. Here, we expected that if attitude roots were discernible among argument endorsements, those clusters of argument endorsements would relate to a specific psychological determinant of vaccine hesitancy. That is, a set of different arguments that invoke conspiracies should be preferentially endorsed by people who tend to view the world through a conspiratorial lens, whereas arguments that emerge from a libertarian lens should be preferentially endorsed by free-market advocates, and so on. However, as many of these psychological constructs may themselves be intercorrelated^15,24,27,28^, this could hinder the ability to discern specific associations of one psychological construct with its expected argument endorsements. Nonetheless, each psychological construct should at minimum be associated with argument endorsement strength.

## Results

### Factor structure of argument endorsements

We first analysed the internal structure of participants’ endorsement of the anti-vaccination arguments (66 in Sample 1, 33 in Sample 2). We started exploring both datasets through Exploratory Factor Analysis (EFA) with maximum likelihood estimation and promax rotation. In the first sample, parallel analysis suggested to retain 3 factors. However, the 3-factor solution displayed numerous cross-loadings and the factors were not interpretable from a theoretical point of view, which suggested that the 1-factor solution, which displayed acceptable item loadings in all cases ($> 0.34$), would be preferable. In the second sample, a parallel analysis suggested a 2-factor solution, with religious concerns grouped into a separate factor. As in Sample 1, a 1-factor EFA solution was viable, with all loadings $> 0.36$.

We then used pre-registered Confirmatory Factor Analyses (CFA) to evaluate 3 models compatible with the taxonomy of anti-vaccination arguments^21^: a 1-factor model, an 11-factor model in which all the attitude roots were represented as different latent variables, and a 7-factor model in which 4 pairs of thematically related attitude roots were collapsed into combined factors: (1) conspiracist ideation and distrust, (2) religious and moral concerns, (3) fear and phobias and distorted risk perception, and (4) perceived self-interest and reactance. Parameters were estimated by maximum likelihood method, which allows the calculation of the commonly used criteria for acceptable goodness-of-fit: Comparative Fit Index (CFI) and Tucker-Lewis Index (TLI) close to 0.90 or above, Root Mean Square Error of Approximation (RMSEA) close to 0.08 or below, and Standardised Root Mean Square Residual (SRMR) close to 0.05 or below^29^. The 7-factor and 11-factor models were not acceptable due to poor fit indices in both samples and because they implied mathematically impossible variance-covariance matrices. We theorise that this is attributable to extremely high correlations among items that were designed to measure distinct roots. We also explored statistical approaches that are more tolerant under conditions of substantial intercorrelation between latent variables, such as confirmatory bi-factor models or exploratory structural equation modeling, and those were also found to be unsatisfactory. The 1-factor model was unproblematic in both samples. The results are displayed in Table 2. Sample 1 uses all 66 items, whereas Sample 2 uses the substantially narrowed item pool described above. Our results confirm the notion that people who are opposing vaccinations will tend to endorse any and all anti-vaccination arguments within the taxonomy.

**Table 2:** Fit indices of the 1-factor models using confirmatory factor analysis.

Sample	$\chi ^{2}$	TLI	CFI	RMSEA (CI)	SRMSEA
1	9266.35 (*p* < 0.001)	0.74	0.75	0.07 (0.072-0.074)	0.08
2	2225.86 (*p* < 0.001)	0.91	0.91	0.08 (0.074-0.080)	0.04

### Associations of anti-vaccination argument endorsement

Following our pre-registered hypotheses, we next examined the relationship between argument endorsements and the assays of the attitude roots. As shown in Table 3, 11 out of 13 of the measured psychological constructs in Sample 2 were significantly correlated in the expected direction with argument endorsements drawn from the target attitude root. The constructs were also associated with total endorsements of all the arguments, is in line with the unidimensional structured revealed by the factor analyses. These correlations remained significant, with similar effect sizes, when controlling for age, gender, education, and political orientation (see Supplementary Information, Table S4). We interpret these results as partial support for our hypotheses. Although we found 11 of the 13 hypothesised correlations, these associations are not exclusive to the target attitude, but rather extend to the rest of attitude roots, which, again, suggests a monological belief system among those who endorse anti-vaccinations arguments.

Two exceptions, Trait Fear and Prosocial Behavioural Intentions, did not correlate significantly with argument endorsements from the target attitude root—and Prosocial Behavioural Intentions did not correlate at all with overall argument endorsements. However, these constructs were also not significantly associated with their related dimensions of the 5C scale—confidence (*r* = − 0.02) and collective responsibility (*r* = − 0.03), respectively. By contrast, all other psychological constructs were significantly correlated to the 5C measures, indicating that these were predictive of the vaccine hesitancy determinants. We are inclined to attribute this lack of association for the two exceptions to the scales used to measure Trait Fear and Prosocial Behavioural Intentions, which may be too general and, consequently, do not reflect the specific psychological processes related to healthcare or vaccination.

In addition, average argument endorsement correlated positively with the 5C subscales Constraints, Complacency, Calculation, Collective, as well as negatively with the Confidence subscale^30^, as shown at the bottom of Table 3. Greater endorsement of the arguments therefore indicated greater vaccine hesitancy. We also found in exploratory hierarchical linear regressions (available in Supplementary Information, Table S5) that argument endorsements had incremental validity in predicting the psychological constructs, over and above the 5C items.

**Table 3:** Zero-order correlations of argument endorsements with the predicted psychological constructs and the 5C scale.

Psychological construct	Argument endorsements	
	Of target attitude root	Of all arguments
Conspiracy mentality	0.60***	0.59***
General distrust	0.19***	0.20***
Pseudoscientific beliefs	0.47***	0.44***
Centrality of religion	0.33***	0.22***
Moral absolutism	0.13***	0.14***
Trait fear	0.07	0.09*
Prosocial behavioral intentions	0.01	0.04
Alternative epistemology	0.50***	0.55***
General reactance	0.22***	0.24***
Free market ideology	0.27***	0.30***
Traditionalism	0.17***	0.23***
Populism	0.45***	0.43***
Perceived vaccination risk	0.68***	0.68***
5C scale		
Confidence	–	− 0.83***
Constraints	–	0.43***
Complacency	–	0.79***
Calculation	–	0.14***
Collective	–	0.71***

All correlations are Spearman’s based on the Kolmogorov-Smirnov test. *p < 0.05; **p < 0.01; ***p < 0.001.

We also observed that the psychological constructs were significantly correlated with each other, albeit to a lesser extent than the argument endorsements (correlation coefficients among the psychological constructs can be found in Supplementary Information, Table S6). This prompted us to further examine how the psychological constructs might overlap among individuals through an exploratory analysis of psychological profiles at the participant level.

### Psychological profiles of anti-vaccination argument endorsement

We next used Latent Profile Analysis (LPA) to identify different profiles of participants in Sample 2 (*n* = 590) based on their responses to the psychological constructs. LPA is a variant of latent class analysis that allows the use of continuous variables. LPA is a person-centered analytic tool that offers a classification of each participant in the most probable profile based on a set of observable variables, rather than classifying the variables^31^. A range of indices determine the most appropriate number of latent profiles: Akaike Information Criterion (AIC), Bayesian Information Criterion (BIC), sample-size adjusted BIC (SABIC), and a measure of entropy. Lower values for AIC, BIC, and SABIC indicate greater fit. For entropy, values above 0.80 denote reliable separation of profiles.

**Table 4:** Fit of latent profile model for psychological constructs in Sample 2.

No. of profiles	AIC	BIC	SABIC	Entropy
1	21,806	21,919	21,837	–
2	20,689	20,864	20,737	0.85
3	20,354	20,591	20,419	0.84
4	20,193	20,491	20,275	0.83
5	20,094	20,454	20,193	0.85
6	19,986	20,407	20,102	0.83
7	19,934	20,416	20,066	0.85
**8**	**19,847**	**20,390**	**19,996**	**0.85**
9	19,831	20,435	19,997	0.85
10	19,795	20,461	19,978	0.83

Selected model in bold.

**Figure 1:**
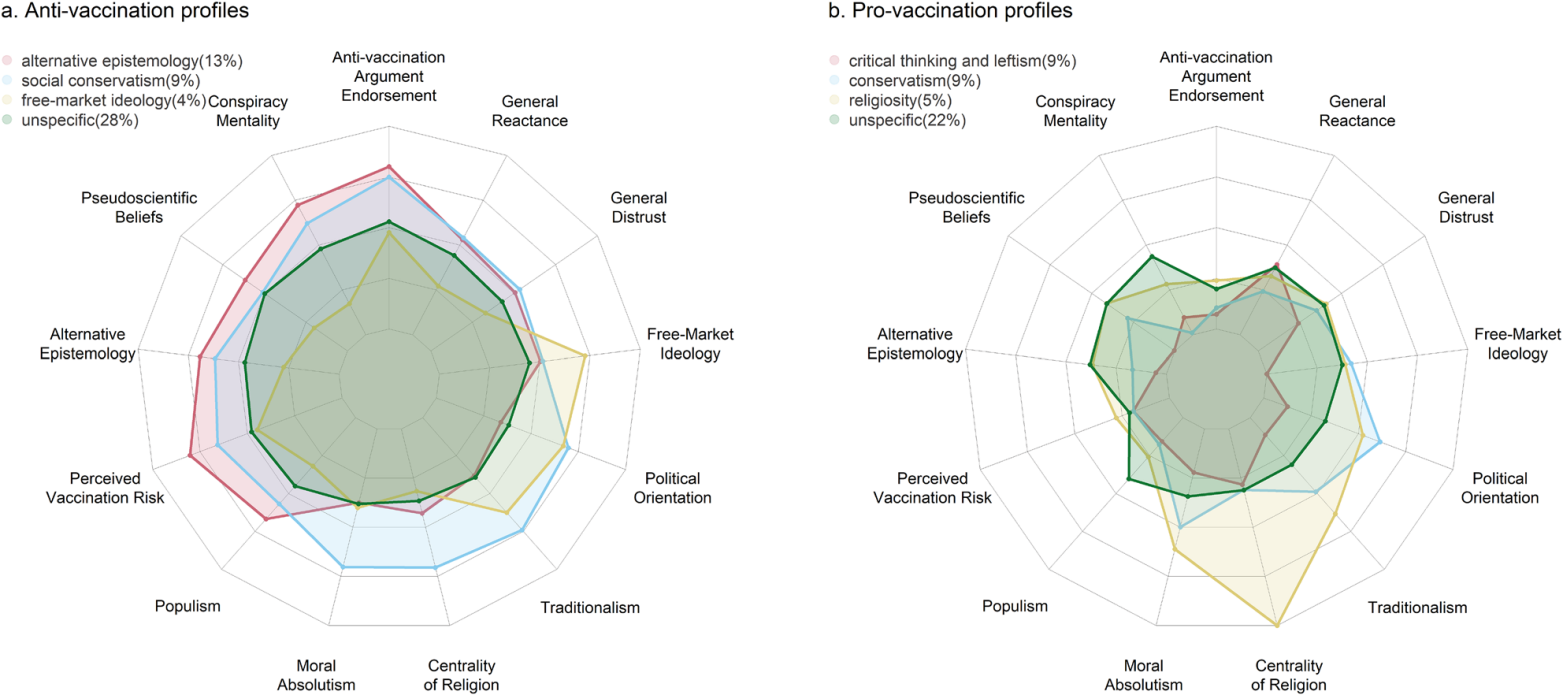
Anti- and pro-vaccination profiles identified in a latent profile analysis using 13 psychological constructs associated with endorsement of anti-vaccination arguments.

We included in the LPA the 11 psychological constructs that were associated with argument endorsements at *p* < 0.001 (see Table 3). To distinguish between pro-vaccination and anti-vaccination profiles, we included the average of participants’ endorsements for all arguments to classify their overall vaccination attitude. We additionally included Political Orientation as it was positively associated with argument endorsement (*r* = 0.16, $\textit{p} < 0.001$). Table 4 shows the fit indices of models with 1–10 profiles. We selected the 8-profile model, which exhibited the lowest BIC and a high entropy. We used the scores of each profile in endorsement of anti-vaccination arguments to define their respective attitude toward vaccinations (i.e., if they were below or above the mean). Four profiles were characterised as pro-vaccination and four as anti-vaccination (see Fig. 1).

Two of the identified profiles (“unspecified” pro- and anti-vaccination) did not show any distinctive association and exhibited moderate levels of pro- and anti-vaccination attitudes. The remaining six profiles had distinctive and consistent patterns. Among the anti-vaccination profiles, we found an “alternative epistemology” profile characterised by a combination of anti-scientific beliefs and epistemology, with this group being particularly prone to endorsing anti-vaccination arguments. A second “social conservatism” profile was characterised by high religiosity, moral rigidity, and traditionalism. The last, “free-market ideology”, anti-vaccination profile was predominantly characterised by its strong endorsement of this ideology. In contrast, among the pro-vaccination profiles we found a “critical thinking and leftism” profile characterised by its opposition to anti-scientific beliefs, alternative epistemology, and conservatism, and this profile appeared especially resistant to anti-vaccine arguments. The second “conservatism” profile was characterised by moderate conservative ideology and lower General Reactance and Alternative Epistemology than their anti-vaccination counterparts—which suggests an ideological profile less susceptible to politically motivated reasoning. The final pro-vaccination profile was characterised by its religiosity—which is in line with the context-dependent relationship between religiosity and vaccine hesitancy^32^.

## Discussion

We investigated the psychological factors, or “attitude roots”, motivating contrarian views regarding vaccines and the endorsement of these arguments. We selected attitude roots based on a taxonomy of anti-vaccination arguments^21^, and operationalised them in two ways. First, we selected a group of prototypical anti-vaccination arguments to represent themes from each attitude root in the original taxonomy. Second, we selected psychological construct measures that were conceptually aligned with the attitude roots. The analyses of these two different attitude root representations give rise to a complex overall picture of how people may sustain anti-vaccination attitudes. Individuals who scored higher on the psychological antecedents of vaccine hesitancy and the psychological construct representations of the attitude roots endorsed all arguments against vaccines more strongly. These individuals further clustered into four identifiable “profiles” based on the psychological constructs.

In two UK samples totalling 1250 responses, we found that endorsements of arguments selected to represented distinct attitude roots were so correlated that it constrained our ability to fit the proposed 11-factor structure model. We were thus unable to confirm any preferential endorsement patterns among arguments representing different attitude roots. This finding may seem at odds with previous work that was able to classify documented arguments that people had put forth against vaccination, where a computational model trained on scholarly analysis of the arguments could successfully predict the attitude roots of new arguments from a different domain^21^. However, it is important to distinguish endorsement of arguments that one may encounter from the expression of arguments that one may produce. When presented with an arguments against vaccines, an individual who is strongly negative about vaccination may strongly endorse any argument simply because it is consistent with their attitude. This tendency to endorse arguments that support one’s existing perspective has been documented in a family of cognitive biases, such as “belief bias” and “myside bias”, where people evaluate evidence and accept conclusions in a manner that is biased towards their prior opinions and attitudes^33^. Reasoning research in particular has shown that attitude-consistent conclusions are believed more than attitude-inconsistent ones, especially when these conclusions relate to ideological and political issues (e.g.,^34–36^). For example, participants’ prior attitudes towards abortion impeded their ability to discern flawed reasoning when the conclusions supported their abortion position^36^. People also tend to endorse otherwise identical information more if it supports one’s political beliefs rather than challenges them^37^. In extreme circumstances, endorsing one argument can even act as support for the endorsement of others towards the same conclusion, forming a monological belief system that is commonly seen within conspiracist ideation^26^. Indeed, beliefs in distinct conspiracy theories correlate highly with one another, even when those theories are contradictory or fictitious^25,38–40^. The motivations for one’s anti-vaccination attitude may thus be captured by one (or several) arguments that one might personally express, but endorsing other attitude-consistent arguments can serve to support, and perhaps strengthen, one’s overall position.

The motivations for endorsing anti-vaccination arguments may instead need to be captured by other measures. We included psychological construct measures in Sample 2 to assess the associations of these assays of the attitude root to argument endorsements. With the exception of trait fear and prosociality (which also did not predict the relevant 5C vaccine hesitancy determinant), each psychological construct was not only associated with argument endorsements from the target attitude root, but also argument endorsements from other attitude roots. In other words, there was an overall tendency for those with high levels of those psychological factors to give stronger endorsements of anti-vaccination arguments in general. The effect sizes of the significant correlations varied from *r* = 0.14–0.68, so some of these correlations were only weak ones. However, these correlational effect sizes, and their directions, generally match with those found in previous research regarding the relationship of the psychological constructs to other vaccine hesitancy measures^19,20,23,41–45^. It is important to clarify that our study related the psychological assays for the attitude root to both argument endorsement strength and vaccine hesitancy determinants (measured by the 5C scale^30^), as vaccine hesitancy has historically been ambiguously defined, with researchers conceptualising the term in varying ways, from cognition and affect to decisions and behaviour^46^. Our findings thus link this comprehensive set of psychological constructs to their role in motivating cognition about vaccines, which themselves predict previously-validated behavioural determinants of vaccine hesitancy. Where a psychological construct predicted vaccine hesitancy in the literature, it also predicted argument endorsement to a similar extent. In both cases, some of these correlations were weak, which may reflect a difficulty in selecting the right measures to capture the latent psychological variables. More likely, it indicates that people can possess various overlapping motivations, each of which contribute in part to their attitudes and endorsements.

It was difficult to determine unique contributions of attitude roots to anti-vaccination argument endorsement strength. We had expected some level of clustering, where certain attitude roots should be more strongly associated than others, which was captured by the 7-root model that collapsed these attitude roots. For example, conspiracist ideation, which is characterised by a high level of distrust in the “official” narrative^47^), should be strongly associated with distrust, even if it is distinct in that not all distrust in vaccination involves a belief in a conspiracy theory. We observed not just these expected clusters, but also a more complex pattern of inter-correlations among all the psychological constructs. To some extent, this is supported by past research—for example, conspiracist mentality is correlated with right-wing social conservatism^27^, free-market ideology^15^, distrust of official information sources^24^, and paranormal and pseudoscientific beliefs^28^. However, our study is the first to systematically investigate this many potential attitude roots and assess their overlap.

Making the case for potential overlap among attitude roots^18^, proposed that multiple roots could sustain an individuals’ attitudes and “in combination they could be more powerful than if one were to operate individually.” Our latent profile analysis provided some evidence for this proposal, as we were able to identify four distinct clusters of participants in Sample 2 who tended to endorse anti-vaccination arguments. These profiles were primarily characterised by various elements of anti-science beliefs and ideological partisanship. We consider below how the psychological clusters we found could be targeted for vaccine communication interventions, which future research may wish to build on.

One profile that displayed average scores across all constructs and less strong argument endorsements suggested that this might be an “on-the-fence” group who could be more amenable to informational interventions such as prebunking and debunking^48^. Because no one psychological construct distinguishes this group, it may be worth considering broader spectrum interventions that do not target specific misinformation content but warn against strategies marking out misinformation^49^. Techniques such as Motivational Interviewing may also be useful, as it encourages healthcare professionals engaging with patients who are uncertain about a certain behaviour (including vaccination) to explore their motivations for it and guide patients towards acceptance^50^.

Conversely, the psychological profile with the strongest total endorsement of anti-vaccination arguments and perception of vaccination risks encompassed individuals who may seek to justify their hesitancy through a combination of anti-scientific doctrines (i.e., conspiracy and pseudoscientific theories) and an alternative epistemology that undermines normative epistemological principles such as the primacy of scientific evidence. These attitude roots may also reinforce one another inasmuch as a relativistic epistemology facilitates the adoption and promotion of anti-scientific conceptions^51^. This group would be highly likely to resist correction that are based on a shared acceptance of facts and evidence. Communication with this group would gain most from first establishing a common ground for further discussion before attempting to correct any misconceptions. Consider, for instance, an individual who holds strong beliefs about the effectiveness of alternative medicinal products. Rather than arguing that there is insufficient evidence for alternative medicine and overwhelming evidence for vaccination, it may be more productive to acknowledge that one can reap benefits from different types of therapies—but these are in addition to rather than instead of vaccination.

The final profiles were characterised by high levels of social conservatism, with the smaller of these profiles distinguished by an additional stronger belief in free-market ideology—reflecting a divergence in conservative ideology on social and economic issues^52,53^. A vaccine communication strategy for these groups could focus on how vaccination is not at odds with their belief systems. For example, information and corrections will likely be trusted more and seen as compatible with beliefs if they come from authoritative sources within one’s religion, tradition, or community group^54^. Identifying benefits that align with existing worldviews may also be important, for example, positioning vaccination as an individual choice to gain its protective benefits for oneself (as opposed to benefiting society) would be in line with a neoliberal ideology that prizes individualism and deregulation^55^.

Our research also has a few limitations that future research may also wish to address. As a correlational study, although it is reasonable to posit that psychological factors drive anti-vaccination belief, our study design does not allow us to draw such a causal conclusions from the observed significant associations. We also focused on soliciting endorsements of arguments from different roots rather than having participants express reasons for rejecting vaccinations. While this method was necessary to allow us to determine the factor structure of the endorsements and their correlations with the psychological constructs, it could be good for future research to investigate if the psychological constructs are related to the types of arguments people might choose to express against vaccination.

We are also cautious about the generalisability of the psychological profiles in our UK sample, and recommend that future research address whether these profiles are present in other countries and cultures. The associations between some of the psychological constructs and vaccine hesitancy may differ, for example, reactance was previously found to predict anti-vaccination attitudes in the UK (*r* = 0.33) but not in Japan (*r* = 0.09)^20^. Specifically, associations around worldview and politics will likely be sensitive to different cultural contexts, as the relationship between social and economic conservatism is characteristic of developed countries^56,57^.

In sum, our work contributes data covering a comprehensive set of psychological factors associated with vaccine hesitancy, and, specifically, its cognitive manifestation as endorsements of anti-vaccination arguments. We found that these endorsements exhibit a monological response pattern, with high inter-correlations, but the psychological factors that predicted argument endorsement strength clustered into distinct psychological profiles. These indicate that two key motivators of anti-vaccination belief relate to anti-scientific conceptions and political polarisation, which may require different communication strategies to tackle.

## Methods

Before data collection, the study was approved by the University of Bristol School of Psychological Science Research Ethics Committee (references: 10309 and 10708) and the study methods and planned analyses were pre-registered. The pre-registration, study materials, data, and analysis scripts to derive our reported results are shared on the Open Science Framework: https://osf.io/27f5u/. All study methods were performed in accordance with the relevant guidelines and regulations approved by the Research Ethics Committee. Informed consent was obtained from all participants prior to their participation in the study.

### Participants

We recruited 1250 participants (Sample 1: *n* = 660; Sample 2: *n* = 590) from the UK via Prolific, who were paid at a rate of $\pounds$9/h. We determined sample sizes based on a recommended ratio for performing factor analyses of at least 10 participants per measured anti-vaccination argument^58^. For Sample 1, we pre-selected participants who had stated they either felt negatively or neutral towards the COVID-19 vaccine in a Prolific screening question. This was to ensure we would have enough participants who would endorse the anti-vaccination arguments to enable a factor analysis. For Sample 2, we did not apply this pre-selection filter because we aimed to assess a wider range of attitudes and psychological characteristics that would enable the correlational analyses and allow us to build profiles of individuals with pro- and anti-vaccination views. This also meant that Sample 2 should be less prone to monological thinking than Sample 1. In both cases, we obtained a balanced distribution of gender and political leanings.

At the end of each data gathering process, participants provided demographic information. Participants in Sample 1 were 50% male, 49% female (1% did not identify with either gender), with ages ranging between 18-84 years (*M* = 38.36, *SD* = 12.13). Participants in Sample 2 were 49% male, 51% female, with ages ranging between 18–85 years (*M* = 43.10, *SD* = 14.12). In both samples, 48% had at least a Bachelor’s degree, and there was a normal distribution across political leanings on an 11-point scale representing the left-right political spectrum^27,59^ (Sample 1: *M* = 5.74, *SD* = 2.33, skewness = 0.06, kurtosis = − 0.37; Sample 2: *M* = 5.83, *SD* = 2.40, skewness < 0.01, kurtosis = − 0.62).

### Measures

#### Anti-vaccination arguments

We assessed participants’ endorsements of anti-vaccination arguments using a methodology similar to that used in research investigating conspiracist beliefs (e.g.,^60^. Participants endorsed arguments by indicating how much they agreed with each argument on a 7-point Likert scale (1: strongly disagree, 7: strongly agree). All arguments and their mean endorsement ratings in our two samples are available in Supplementary Information (Table S2).

In the first sample, participants rated their endorsement of 66 prototype anti-vaccination arguments that were spread evenly across the 11 attitude roots. All but four of the arguments were prototypical arguments identified in Fasce et al.’s^21^ taxonomy. The remaining four were created for the purposes of this study to ensure no root had disproportionately fewer arguments for analysis than the others. The levels of skewness and kurtosis of most of the arguments assessed in Sample 1 were within the usual thresholds for a normal distribution (+ 2/− 2). Only three arguments (the second and fourth of religious concerns, and the second of reactance) exhibited a kurtosis slightly above the threshold (2.71, 2.12, and 2.47, respectively). These 66 arguments showed high internal consistency ($\alpha$ of the six arguments within each attitude root ranged from 0.67 to 0.91; total $\alpha$ = 0.98).

We used a 11 exploratory bi-factor latent variable models (i.e., with the Schmid-Leiman transformation^61^) of each set of argument endorsements in Sample 1 to identify arguments most diagnostic for their attitude root to use in Sample 2. Specifically, we selected the three arguments for each attitude root that were most saturated with the general factor. Explained Common Variance of the general factors ranged from .43 (moral concerns) to .86 (epistemic relativism), with a median of .74, suggesting that most target roots were relatively unidimensional. The three items with general factor loadings > .60 for each target root were retained for Sample 2, resulting in 33 anti-vaccination arguments (the list of selected arguments and their respective mean endorsements can be found in the Supplementary Information, Table S2). This shortened the overall questionnaire while maintaining a minimum of three items per attitude root required for a confirmatory factor analysis on these data. Participants in Sample 2 rated their endorsement of these 33 arguments. The parameters of skewness and kurtosis of almost all the 33 anti-vaccination arguments included in the second sample were within the thresholds of normality, except for the second and fourth arguments of religious concerns, which showed higher levels of kurtosis (3.55 and 2.17, respectively). The total $\alpha$ of the 33 anti-vaccination arguments was 0.98, with the internal consistency of the attitude roots ranging from 0.75 to 0.91. We found very similar patterns of argument endorsement between the two samples, indicating that the three items per root chosen for the second sample remained representative of that root.

#### Psychological constructs

In Sample 2, we also collected data on participants’ responses to 13 previously validated measures of psychological constructs that were selected as independent assays of the 11 attitude roots. Participants also responded to the short version 5C scale^30^, which composes five items for each of its dimensions, to be used independently: Confidence, Constraints, Complacency, Calculation, and Collective (responsibility). This gave us a general measure of the psychological antecedents of vaccination behaviour and, consequently, vaccine hesitancy, which provided further validation that the argument endorsements were associated with vaccine hesitancy (see Table 3). We summarise the psychological construct measures here, with relevant statistics in the Supplementary Information (Table S3).

- *Conspiracy Mentality*. Conspiracy Mentality Questionnaire^62^, five items measuring generic conspiracy beliefs.
- *General distrust*. (Dis)trust Scale^63^’s, eight items measuring general trust towards other people (reverse coded for distrust).
- *Pseudoscientific beliefs*. Short-form Pseudoscientific Belief Scale^28^, eight items measuring general unwarranted beliefs falsely presented as scientific.
- *Free market ideology*. Free-market Endorsement Scale^15^, five items measuring economic conservatism through the promotion of *laissez-faire* capitalism and private enterprise.
- *Traditionalism*. Four items from the conventionalism factor of the Aggression-Submission-Conventionalism Scale^64^ that express traditionalism (as opposed to respect for social norms).
- *Populism*. Three items with the highest factor loading on the Populist Attitudes Scale^65^, defined as a political attitude with three core features: sovereignty of “the people”, opposition to the elite, and the Manichean division between “good” people and “evil” elites.
- *Centrality of religion*. Centrality of Religion Scale^66^, five items measuring salience of religious meanings in personality.
- *Moral absolutism*. Moral Absolutism Scale^67^, six items measuring desire for certainty in the moral domain. An additional measure of moral exporting was discarded due to poor internal consistency.
- *Trait fear*. Six items with factor loadings> 0.70 from the Trait Fear Scale^68^, measuring self-reported variations in fear and fearlessness.
- *Perceived vaccination risk*. Following^30^, we asked participants to rate the risk of four diseases (Covid-19, influenza, measles, and HPV) and the risk of their respective vaccines. To calculate the likelihood and magnitude of perceived risk of vaccination in comparison to that of vaccine-preventable diseases, we subtracted the risk of vaccines scores from the risk of disease scores.
- *Prosocial behavioral intentions*. Prosocial Behavioral Intentions Scale^69^, four items measuring participants’ general prosociality in common situations.
- *Alternative epistemology*. Epistemic Beliefs Scale^51^, 12 items with 3 sub-factors measuring epistemic beliefs. The first factor measures reliance on intuition for factual beliefs, the second reflects conviction that facts are politically constructed, and the third measures importance of consistency between empirical evidence and beliefs. The third factor was reversed to denote rejection of evidence and, subsequently, calculate a total score in alternative epistemology.
- *General reactance*. A condensed version of the Hong Psychological Reactance Scale^70^ used in^20^, with five items measuring motivation to reject consensus views as part of a nonconformist identity.

To assess the convergent and discriminant validity of the argument endorsements in relation to their associated psychological constructs, we pre-registered the following predictions based on previous findings on anti-vaccination arguments and vaccine hesitancy:

1. Endorsement of conspiracist ideation arguments would be positively correlated with Conspiracy Mentality (correlation coefficients ranging from 0.14 to 0.52^15,20,23,24,71^).
2. Endorsement of distrust arguments would be positively correlated with General Distrust (correlation coefficients ranging from 0.20 to 0.58^19,24,72^).
3. Endorsement of unwarranted belief arguments would be positively correlated with Pseudoscientific Beliefs (correlation coefficients ranging from 0.24 to 0.50^24,41^).
4. Endorsement of worldview and politics arguments would be positively correlated with Free Market Ideology (correlation coefficients ranging from 0.22 to 0.24^41,42^), Traditionalism (correlation coefficients ranging from 0.14 to 0.38^20^), and Populism (correlation coefficients ranging from 0.72 to 0.79^43^).
5. Endorsement of religious concern arguments would be positively correlated with Centrality of Religion (*r* = 0.18^42^).
6. Endorsement of moral concern arguments would be positively correlated with Moral Absolutism (no correlation coefficient previously reported^44,73^).
7. Endorsement of fear and phobia arguments would be positively correlated with Trait Fear (correlation coefficients ranging from 0.09 to 0.50^20,74,75^).
8. Endorsement of distorted risk perception arguments would be positively correlated with Perceived Vaccination Risk (correlation coefficients ranging from 0.11 to 0.86^30^).
9. Endorsement of perceived self-interest arguments would be negatively correlated with Prosocial Behavioral Intentions (no correlation coefficient previously reported^76–83^).
10. Endorsement of epistemic relativism arguments would be positively correlated with Alternative Epistemology (correlation coefficients ranging from 0.17 to 0.20^45,84,85^).
11. Endorsement of reactance arguments would be positively correlated with General Reactance (correlation coefficients ranging from 0.14 to 0.47^20,24^).

## Supplementary Information

Supplementary Information.

## Data Availability

All datasets used in this article are publicly available at https://osf.io/27f5u/.

## Code availibility

All source code used in this article is publicly available at https://osf.io/27f5u/.
