# Supplementary material for: Psychological profiles of anti-vaccination argument endorsement
Source: Sci Rep. 2023 Jul 17;13:11219. doi: 10.1038/s41598-023-30883-7 (PMC10352341; doi:10.1038/s41598-023-30883-7)
Supplement: Supplementary file 1 — Supplementary Information. [file 41598_2023_30883_MOESM1_ESM.pdf]

Psychological profiles of anti-vaccination argument endorsement

Supplementary Information

Participants

Table S1 provides a full breakdown of participant demographics for participants in the two samples described in the study.

Table S1. Participant demographics in Samples 1 and 2.

	**Sample 1 (n = 660)**		**Sample 2 (n = 590)**	
Age (in years)	Range: 18-84; *M* = 38.36, *SD* = 12.13		Range: 18-85; *M* = 43.10, *SD* = 14.12	
Gender identity:				
Male	329	50%	288	49%
Female	327	49%	299	51%
Non-binary, both or self-defined	4	1%	3	< 1%
Highest education completed:				
No formal qualifications	8	1%	11	2%
High school or GCSEs	117	18%	112	19%
Sixth form college or A level equivalent	214	32%	183	31%
Bachelor degree or equivalent	240	36%	208	35%
Master’s degree	66	10%	63	11%
Doctorate or professional degree	9	1%	13	2%
Did not state	6	1%	0	0%
Employment status				
Full-time	360	55%	267	45%
Part-time	143	22%	122	21%
Not in paid work	124	19%	169	29%
Other or did not state	33	5%	32	5%
COVID-19 vaccine opinion				
Against	214	32%	193	33%
Neutral / NA	446	68%	202	34%
Positive	-	-	195	33%
Alignment on political spectrum (see also Figure S1)	*M* = 5.74; *SD* = 2.33; skewness = 0.06; kurtosis = -0.37		*M* = 5.83; *SD* = 2.40; skewness < 0.01; kurtosis = -0.62	

Figure S1. Distribution of samples along the political spectrum.

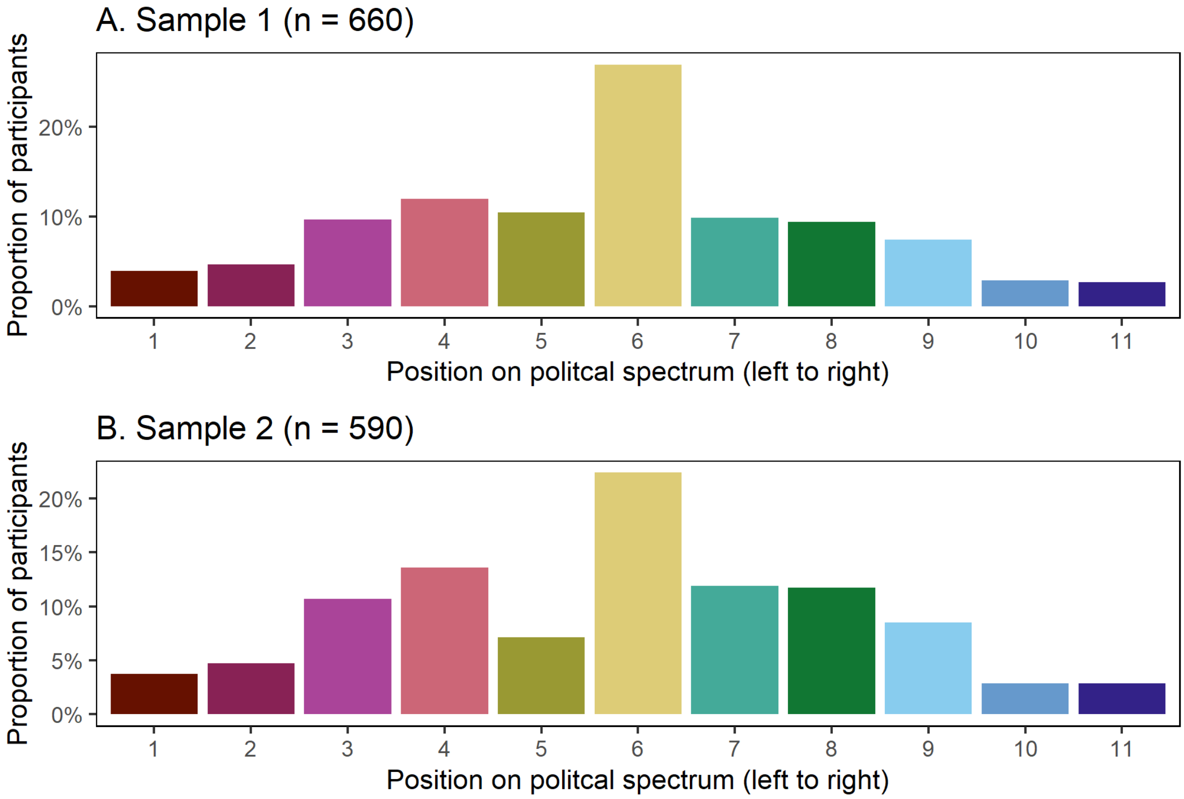

Mean endorsement of anti-vaccination arguments

Table S2 shows the anti-vaccination arguments used in the study, grouped by their attitude root, and the mean and standard deviations of endorsements received from participants in each sample for each argument.

Table S2. Arguments from 11 attitude roots, participants' endorsement for each one, and item loadings in two samples.

Attitude root	Argument	Mean (SD) endorsement		Exploratory bi-factor model loadings in Sample 1	11-factor CFA loadings in Sample 2
		Sample 1 (n = 660)	Sample 2 (n = 590)		
Conspiracist ideation	The authorities are lying and covering up important information about the vaccine.	4.37 (1.81)	3.72 (2.19)	0.77	0.90
	"Big Pharma" is colluding with the medical authorities to profit from people getting vaccinated.	5.00 (1.73)	-	0.66	-
	Some vaccines contain microchips that will be used to control us.	1.80 (1.28)	-	0.45	-
	To get us vaccinated, medical authorities are spreading fear about diseases that do not exist or are fabricated.	3.92 (1.92)	3.41 (2.15)	0.71	0.85
	Vaccination campaigns are targeted at weakening disadvantaged groups, minorities, and poor countries.	3.32 (1.76)	3.04 (1.87)	0.64	0.75
Distrust	When it comes to vaccines, doctors do not know what they are doing and do not care about patients.	3.00 (1.58)	-	0.64	-
	Research on vaccine safety is based on biased or incomplete data.	4.36 (1.75)	3.76 (1.99)	0.74	0.86

Attitude root	Argument	Mean (SD) endorsement Sample 1 (n = 660)	Sample 2 (n = 590)	Exploratory bi-factor model loadings in Sample 1	11-factor CFA loadings in Sample 2
	The alleged scientific studies on vaccines are based on discredited theories and flawed methodologies.	3.43 (1.63)	-	0.71	-
	Medical authorities are overreacting, with vaccines being recommended for every minor illness now.	4.27 (1.88)	3.77 (2.12)	0.72	0.86
	Information from “Big Pharma” about vaccines is not to be trusted.	4.66 (1.63)	-	0.72	-
	Healthcare authorities, politicians, and governments are corrupt and profit from vaccinations.	5.07 (1.71)	-	0.67	-
	There is not enough safety testing, and no one is liable if someone is harmed by the vaccine.	5.00 (1.78)	4.25 (2.18)	0.75	0.84
	Powerful groups oppress disadvantaged groups and genders by imposing vaccination.	3.92 (1.84)	-	0.66	-
	People should do their own research and decide rather than following so-called “experts”.	4.68 (1.75)	-	0.58	-
Unwarranted beliefs	Vaccines are not as good as traditional and natural remedies, such as homeopathy, which have a similar record of healing and no side effects.	3.16 (1.73)	-	0.75	-
	Vaccines interfere with the body’s natural immunity, which provides a better defense against the disease	4.08 (1.72)	3.61 (2.01)	0.82	0.82
	People are being offered too many vaccines nowadays, and this will overload their immune systems.	4.22 (1.84)	3.78 (2.12)	0.81	0.91

Attitude root	Argument	Mean (SD) endorsement		Exploratory bi-factor model loadings in Sample 1	11-factor CFA loadings in Sample 2
		Sample 1 (n = 660)	Sample 2 (n = 590)		
	Instead of vaccines, people should improve environmental factors like good hygiene, healthy lifestyles, and protective measures against the disease.	4.68 (1.66)	-	0.72	-
	Scientists are still debating the benefits of vaccination, and the science is not settled.	4.61 (1.74)	3.96 (2.02)	0.75	0.83
	Vaccines contain viruses and can cause the disease they are supposed to prevent.	3.56 (1.76)	-	0.59	-
	Vaccinated people spread the disease they got vaccinated against, making the healthy and unvaccinated sick.	3.94 (1.87)	-	0.53	-
	It cannot be a coincidence that when vaccination rates increased, so did case rates and death rates.	3.94 (1.85)	-	0.75	-
	The disease will disappear on its own, following a natural cycle.	4.41 (1.59)	-	0.61	-
Worldview & politics	Vaccines are just another way that the scientific elite are widening inequalities and subjugating ordinary people.	3.64 (1.82)	3.02 (1.93)	0.63	0.86
	Vaccinations are an expression of the inappropriate interference of the state in the freedoms of individual citizens.	3.92 (1.82)	3.33 (2.05)	0.63	0.88
	Politicians use vaccinations as strategies to boost their own political agendas at the expense of the common good.	4.93 (1.72)	4.07 (2.1)	0.78	0.77
	Adhering to one's own traditional, cultural rules is more important than following vaccination guidelines.	3.62 (1.73)	-	0.51	-

Attitude root	Argument	Mean (SD) endorsement Sample 1 (n = 660)	Sample 2 (n = 590)	Exploratory bi-factor model loadings in Sample 1	11-factor CFA loadings in Sample 2
	Vaccines are a modern invention and I try to avoid modern technology as much as possible.	2.35 (1.45)	-	0.41	-
Religious concerns	Vaccines contain products that are forbidden by my religion.	1.80 (1.34)	-	0.48	-
	Vaccines interfere with God's will: He will decide if people get the disease or not.	1.84 (1.37)	1.73 (1.26)	0.69	0.52
	People should abide by what religious leaders say against vaccines.	2.22 (1.4)	1.87 (1.27)	0.60	0.40
	The human body was created in God's image, so it is a sin to defile it with unnatural injections.	1.89 (1.36)	1.89 (1.4)	0.75	0.59
	Not allowing religious exemptions to vaccines is discriminatory.	4.44 (2.02)	-	0.36	-
Moral concerns	Vaccines were developed through unethical experimentation.	3.67 (1.58)	3.38 (1.81)	0.54	0.75
	People should not accept vaccines that are produced using tissues from aborted fetuses.	4.45 (1.87)	4.06 (2.05)	0.58	0.61
	Sexual abstention is preferable to taking the human papillomavirus (HPV) vaccine, which would only promote promiscuity.	2.64 (1.57)	-	0.36	-
	Profit-driven vaccination campaigns are immoral.	5.87 (1.43)	-	0.51	-
	It's our moral duty not to rely on vaccines	3.40 (1.69)	-	0.48	-
	Parents who rely on vaccination for their child's health demonstrate poor values.	2.69 (1.64)	2.43 (1.68)	0.52	0.74
Fears & phobias	I worry about experiencing side effects from the vaccine.	5.20 (1.61)	-	0.50	-
	Vaccines are not safe.	3.36 (1.56)	3.04 (1.76)	0.70	0.86

Attitude root	Argument	Mean (SD) endorsement Sample 1 (n = 660)	Sample 2 (n = 590)	Exploratory bi-factor model loadings in Sample 1	11-factor CFA loadings in Sample 2
	Vaccines cause severe injuries and people never recover from them.	3.84 (1.67)	-	0.67	-
	Vaccines contaminate the human body with toxins, heavy metals or viruses that could alter DNA.	3.35 (1.75)	3.09 (1.91)	0.83	0.86
	Vaccines should not be administered to vulnerable people, such as pregnant women, young children or patients with allergies.	4.66 (1.73)	-	0.48	-
	Vaccines overwhelm the immune system, especially when taken in many doses.	4.20 (1.68)	3.77 (1.98)	0.68	0.87
	I'm afraid of needles and fear the vaccination will hurt.	2.15 (1.69)	-	< 0.20	-
Distorted risk perception	Vaccines are unnecessary and not a medical priority.	3.17 (1.63)	2.75 (1.75)	0.71	0.84
	Vaccine-preventable diseases are mild and can be easily treated.	3.55 (1.6)	-	0.59	-
	Vaccinations are not needed if you live in a developed and safe country.	2.73 (1.5)	-	0.69	-
	Vaccines are riskier than the diseases themselves.	3.31 (1.74)	2.97 (1.81)	0.70	0.88
	Vaccinations are unnecessary if you have a strong immune system that protects you from vaccine-preventable diseases.	3.91 (1.84)	3.38 (2)	0.86	0.86
Perceived self-interest	People do not need to be vaccinated as long as herd immunity exists.	3.93 (1.66)	3.35 (1.87)	0.61	0.80
	We should not take vaccines because other people do not take protective measures to stop spreading the disease.	3.13 (1.63)	2.70 (1.66)	0.67	0.75

Attitude root	Argument	Mean (SD) endorsement Sample 1 (n = 660)	Sample 2 (n = 590)	Exploratory bi-factor model loadings in Sample 1	11-factor CFA loadings in Sample 2
	People should look after their own health rather than put themselves or their child at risk to protect others.*	4.60 (1.64)	-	0.56	-
	Instead of vaccinations, people should just self-isolate to stop the spread of disease.*	3.42 (1.74)	-	0.57	-
	People whose jobs allow them to adopt strong preventive measures against diseases should not need to get vaccinated.*	4.11 (1.79)	3.44 (1.89)	0.66	0.80
Epistemic relativism	The “theories” on which vaccines are based are not "objective" or "true" but are a social construction by scientists that is being imposed on other equally valid perspectives.	3.6 (1.64)	3.19 (1.83)	0.70	0.83
	Negative experiences and testimonies of injuries by patients should be prioritized when deciding whether or not to accept vaccination.	4.47 (1.65)	4.28 (1.9)	0.72	0.78
	The vaccination movement does not respect alternative perspectives on health that are more comprehensive and holistic.	4.56 (1.68)	4.04 (1.96)	0.74	0.77
	People are experts on their own bodies so they may legitimately conclude based on their own reading that vaccination is not for them.	4.66 (1.77)	-	0.64	-
	The vaccine does not work for everyone, and we should not take risks with anything that is less than 100% effective.	4.00 (1.82)	-	0.69	-
Reactance	Vaccination campaigns bully and harass people into getting a vaccine.	5.01 (1.81)	4.16 (2.22)	0.71	0.84

Attitude root	Argument	Mean (SD) endorsement Sample 1 (n = 660)	Sample 2 (n = 590)	Exploratory bi-factor model loadings in Sample 1	11-factor CFA loadings in Sample 2
	People should be able to decide what goes into their bodies, so it should be a matter of free personal choice whether someone gets a vaccine	6.15 (1.24)	5.78 (1.63)	0.71	0.52
	We need to resist an authoritarian state that is abusing its power and violating individual rights by telling us to get vaccinated.	4.83 (1.87)	-	0.70	-
	People are getting vaccinated out of ignorance and fear, according to what the nanny state expects of them.	4.66 (1.91)	3.84 (2.22)	0.71	0.89
	Everyone has the right to contract a disease if they want to.*	4.56 (1.7)	-	0.39	-

Note. * denotes arguments that were added to the original taxonomy to generate at least 5 items per root. Endorsements were measured on a 7-point Likert scale (1: strongly disagree - 7: strongly agree).

Psychological constructs measured in Sample 2

Table S3 gives the psychological constructs measured in Sample 2, along with an example item and the means, standard deviations, Cronbach's alpha, and skewness and kurtosis of the distribution in our sample.

Table S3. Summary statistics for 13 psychological constructs measured in Sample 2.

**Target attitude root**	**Psychological construct measure**	**Example item**	***M* (*SD*)**	**α**	**Skewness**	**Kurtosis**
Conspiracist ideation	Conspiracy Mentality Questionnaire (Bruder et al., 2013); 10-pt Likert	I think that many very important things happen in the world, which the public is never informed about.	7.51 (2.07)	0.88	-0.47	-0.26
Distrust	General Trust Scale (Yamagishi, 1988); 10-pt Likert	One should not trust others until one knows them well.	3.37 (0.70)	0.82	-0.25	-0.05
Unwarranted beliefs	Short-form Pseudoscientific Belief Scale (Fasce et al., 2021); 5-pt Likert	Due to well demonstrated biological reasons, negative emotions and unsolved conflicts or traumas increase the probability of having cancer.	2.90 (0.81)	0.83	-0.43	-0.11
Worldview and politics	Free-market Endorsement Scale (Lewandowsky et al., 2013); 7-pt Likert	An economic system based on free markets unrestrained by government interference automatically works best to meet human needs.	3.55 (1.07)	0.81	-0.18	0.31
	Traditionalism: conventionalism factor of Aggression-Submission-Conventionalism Scale	Traditions are the foundation of a healthy society and should be respected.	3.02 (0.93)	0.83	-0.47	-0.04

Target attitude root	Psychological construct measure	Example item	*M* (*SD*)	α	Skewness	Kurtosis
	(Dunwoody et al., 2016); 5-pt Likert					
	Populist Attitude Scale (Akkerman et al., 2014); 5-pt Likert	The politicians in the British parliament need to follow the will of the people.	3.75 (0.83)	0.67	-0.42	-0.21
Religious concerns	Centrality of Religion scale (Huber & Huber, 2012); 5-pt Likert	How often do you take part in religious services?	2.08 (1.09)	0.93	1.11	0.37
Moral concerns	Moral Absolutism Scale (Peterson et al., 2009); 4-pt Likert	There are absolutely clear guidelines about what is good and evil. These always apply to everyone, whatever the circumstances.	2.10 (0.54)	0.75	0.30	0.01
Fear and phobias	Trait Fear Scale (Kramer et al., 2019); 5-pt Likert	I enjoy doing new things that other people are afraid to do.	2.40 (0.69)	0.86	0.02	-0.60
Distorted risk perception	Perceived Vaccination Risk (Betsch et al., 2018); 0-100 visual analogue scale	How risky do you judge an infection with [vaccinations against] COVID-19 to be?	7.77 (49.90)	0.84	0.81	0.61
Perceived self-interest	Prosocial Behavioral Intentions Scale (Baumsteiger & Siegel, 2019); 7-pt Likert	[How likely would you be to] comfort someone I know after they experience a hardship.	6.08 (0.96)	0.80	-1.45	3.06
Epistemic relativism	Alternative Epistemology: Epistemic Beliefs Scale (Garrett & Weeks, 2017); 5-pt Likert	I trust my initial feelings about the facts.	2.95 (0.58)	0.77	-0.25	0.32
		Facts are dictated by those in power				

**Target attitude root**	**Psychological construct measure**	**Example item**	***M* (*SD*)**	**α**	**Skewness**	**Kurtosis**
		Evidence is more important than whether something feels true.				
Reactance	Condensed Hong Psychological Reactance Scale (Hornsey et al., 2018); 5-pt Likert	When someone forces me to do something, I feel like doing the opposite.	3.25 (0.65)	0.62	-0.32	0.11
-	5C vaccine hesitancy scale (Betsch et al., 2018); 7-pt Likert					
	Confidence	I am completely confident that vaccines are safe	3.86 (2.04)	-	-0.01	-1.38
	Constraints	Everyday stress prevents me from getting vaccinated.	2.00 (1.28)	-	1.25	0.75
	Complacency	Vaccination is unnecessary because vaccine-preventable diseases are not common anymore.	2.62 (1.63)	-	0.87	-0.03
	Calculation	When I think about getting vaccinated, I weigh benefits and risks to make the best decision possible.	5.76 (1.40)	-	-1.64	2.79
	Collective (responsibility)	When everyone is vaccinated, I don't have to get vaccinated, too.	2.94 (1.85)	-	0.71	-0.56

Partial correlations of psychological constructs with argument endorsements

Table S4 gives the partial correlations of each of the measured psychological constructs with total argument endorsement after controlling for age, gender, education, and political orientation.

Table S4. Partial correlations of psychological constructs with anti-vaccination argument endorsement

**Psychological construct**	**Endorsement of anti-vaccination arguments**	
	**of target attitude root**	**of all attitude roots**
Conspiracy Mentality	0.61***	0.61***
General Distrust	0.15***	0.16***
Pseudoscientific Beliefs	0.47***	0.45***
Centrality of Religion	0.28***	0.18***
Moral Absolutism	0.11**	0.12**
Trait Fear	0.10*	0.11**
Prosocial Behavioral Intentions	0.03	0.05
Alternative Epistemology	0.50***	0.56***
General Reactance	0.23***	0.26***
Free Market Ideology	0.26***	0.28***
Traditionalism	0.15***	0.19***
Populism	0.44***	0.44***
Perceived Vaccination Risk	0.72***	0.77***
5C scale		
Confidence	-	-0.82***
Constraints	-	0.41***
Complacency	-	0.77***
Calculation	-	0.17***
Collective	-	0.67***

Note: * = $p < 0.05$; ** = $p < 0.01$; *** = $p < 0.001$

Incremental validity

We conducted a series of hierarchical linear regression analyses to assess the incremental validity of the scale on anti-vaccination arguments in relation to the 5C scale for psychological antecedents of vaccination, a well-established and widely used multidimensional measure of vaccine hesitancy. The 5 items of the 5C scale (Confidence, Constraints, Complacency, Calculation, and Collective) were independently introduced in Model 1. To avoid reporting marginal effects, we used as dependent variables only those measures for which the 5C scale showed substantial predictive power—defined as accounting for at least 10% of the variance in Model 1. Subsequently, we introduced total anti-vaccination argument endorsement in Model 2. Incremental validity was assumed when total argument endorsement showed a significant standardised β and the adjusted R^2 of Model 2 increased in relation to that of Model 1 (ΔR^2). VIF values were between 1.06 and 5.33, and tolerance statistics were between 0.19 and 0.95. We also observed independence of errors (Durbin–Watson between 1.95 and 2.12). As can be seen from Table S5, incremental validity was confirmed in all cases.

Table S5. Incremental validity of anti-vaccination argument endorsement in relation to the 5C scale.

	**Model 1**	**Model 2**		
	R^2	β	R^2	ΔR^2
Conspiracy mentality	0.28	0.60***	0.35	0.07
Pseudoscientific belief	0.12	0.72***	0.22	0.10
Alternative epistemology	0.24	0.62***	0.31	0.07
Populism	0.15	0.42***	0.19	0.03
Perceived vaccination risk	0.55	0.61***	0.60	0.07
Attitude towards COVID-19 vaccine	0.61	0.47***	0.66	0.04

Note: Model 1 = 5C scale; Model 2 = Total argument endorsement; * = $p < 0.05$; ** = $p < 0.01$; *** = $p < 0.001$.

Correlations between psychological constructs

Table S6 shows the correlations among the psychological constructs measured in Sample 2.

Table S6. Spearman correlations between measured psychological constructs in Sample 2.

	CM	GD	PB	FM	Trad	Pop	Relig	Moral	Fear	Risk	PBI	AE	General Reactance
**Conspiracy mentality (CM)**		0.30***	0.42***	0.13**	0.05	0.54***	0.11*	0.07	0.10*	0.33***	0.12**	0.50***	0.18***
**General Distrust (GD)**			0.12**	0.10*	0.10*	0.21***	0.03	0.04	-0.05	0.06	-0.14***	0.19***	0.27***
**Pseudoscientific beliefs (PB)**				0.20***	0.17***	0.24***	0.25***	<0.01	0.08*	0.18***	0.15***	0.40***	0.09*
**Free-market ideology (FM)**					0.25***	0.22***	-0.01	0.13**	0.09*	0.28***	0.02	0.20***	0.06
**Traditionalism (Trad)**						0.06	0.26***	0.29***	-0.05	0.11**	0.03	0.17***	-0.07
**Populism (Pop)**							0.03	0.08	0.08	0.30***	0.07	0.39***	0.16***
**Centrality of religion (Relig)**								0.17***	-0.03	0.08*	0.08	0.19***	0.02
**Moral Absolutism (Moral)**									-0.08	0.09*	-0.03	0.14***	-0.02
**Trait Fear (Fear)**										0.10*	0.04	0.09*	0.07
**Perceived Vaccination Risk (Risk)**											0.05	0.35***	0.21***
**Prosocial Behavioral Intentions (PBI)**												0.09*	-0.07
**Alternative Epistemology (AE)**													0.27***
With 5C scale:													
**5C confidence**	-0.51***	-0.17***	-0.28***	-0.25***	-0.18***	-0.37***	-0.15***	-0.10*	-0.02	-0.63***	-0.05	-0.43***	-0.20***
**5C constraints**	0.21***	0.16***	0.20***	0.14***	0.01	0.15***	0.11**	0.09*	0.02	0.23***	-0.12**	0.24***	0.16***
**5C complacency**	0.43***	0.14***	0.34***	0.28***	0.16***	0.31***	0.16***	0.17***	0.08*	0.57***	-0.02	0.45***	0.23***
**5C calculation**	0.11**	0.04	0.05	0.03	0.01	0.17***	0.05	-0.10*	0.08*	0.12**	0.24***	0.02	0.05
**5C collective**	0.34***	0.13**	0.23***	0.22***	0.10*	0.30***	0.14***	0.08*	0.12**	0.52***	-0.03	0.35***	0.21***

Note. Asterisks denote significance at * = $p < .05$, ** = $p < .01$, *** = $p < .001$
